# The Role of the Transcription Factor Foxo3 in Hearing Maintenance: Informed Speculation on a New Player in the Cochlea

**DOI:** 10.1155/2016/1870675

**Published:** 2016-10-13

**Authors:** Patricia M. White

**Affiliations:** Department of Neuroscience, University of Rochester School of Medicine and Dentistry, Box 603, 601 Elmwood Avenue, Rochester, NY 14642, USA

## Abstract

Molecular genetics has proven to be a powerful approach for understanding early-onset hearing loss. Recent work in late-onset hearing loss uses mouse genetics to identify molecular mechanisms that promote the maintenance of hearing. One such gene, Foxo3, is ontologically involved in preserving mitochondrial function. Significant evidence exists to support the idea that mitochondrial dysfunction is correlated with and can be causal for hearing loss. Foxo3 is also ontologically implicated in driving the circadian cycle, which has recently been shown to influence the molecular response to noise damage. In this review, the molecular framework connecting these cellular processes is discussed in relation to the cellular pathologies observed in human specimens of late-onset hearing loss. In bringing these observations together, the possibility arises that distinct molecular mechanisms work in multiple cell types to preserve hearing. This diversity offers great opportunities to understand and manipulate genetic processes for therapeutic gain.

## 1. Introduction

One in eight adults has hearing loss, and the likelihood of hearing loss increases as one's age advances [1]. Environmental insults that damage hearing are well known. For example, hearing loss may develop after exposure to prolonged and excessive noise [2] or to ototoxic drugs such as aminoglycosides [3]. Each individual likely has genetic variation in their susceptibility to these insults [4]. Over a hundred genes that affect hearing loss during development have been identified [5]. In contrast, five genome-wide association studies in humans have implicated only a handful of genes in age-related hearing loss [4, 6–9].

Sensory cells of the mammalian cochlea continuously detect and transmit acoustic information throughout the life of the animal. Outer hair cells amplify acoustic vibrations of the tectorial membrane to promote inner hair cell activation. Inner hair cells detect these vibrations and transmit the information to spiral ganglion neurons via ribbon synapses. Sensory hair cell signaling is powered by differential levels of potassium in separate fluid compartments, called the endocochlear potential. The endocochlear potential is actively maintained by the cells of the stria vascularis, in combination with supporting cells and the cells of the spiral ligament. No regeneration has been reported for lost cells in the adult organ of Corti. Consequently, any cellular losses will persist and accumulate through the lifetime of the mammal.

Animal models have elucidated the cellular pathological sequelae of specific inner ear insults [10]. Noise exposure, particularly loud, low-frequency sounds, can destroy outer hair cells in the basal turn [11], termed noise-induced hearing loss. Noise exposure that induces temporary changes in hearing can simultaneously eliminate high-frequency ribbon synapses [12, 13]. Termed synaptopathy, synaptic losses, can occur even when hair cells are completely preserved [12–14]. Glutamate toxicity is one probable mechanism for synaptic loss after noise exposure [15]. Neuronal loss can follow synaptic loss [12]. Administration of aminoglycoside antibiotics also results in hair cell death and spiral neuron degeneration in humans [16, 17] and other species [18]. In addition, injury to the stria vascularis can degrade hearing ability. Reduced endocochlear potential and concomitant hearing loss have been reported in aged gerbils [19]. Moreover, application of furosemide, a diuretic and inhibitor of the Na-K-2Cl symporter (NKCC2), to the gerbil inner ear both reduces endocochlear potential and induces hearing loss [20, 21].

Given that a limited number of cell types participate in hearing and the fact that ototoxic insults preferentially affect specific structures in animals, one could imagine designing a suite of therapeutics that each addresses individual kinds of damage. For example, one drug could be designed that protects synapses from glutamate toxicity. Another one could restore the endocochlear potential, and a third one could protect or regenerate outer hair cells. For this thought experiment, it is important to consider the cellular pathologies commonly found in human disease.

Qualitative analysis of human postmortem samples reveals cellular pathology similar to animal models. Loss of outer hair cells, especially in the basal turn, is a common finding [14], along with strial atrophy [22]. Individuals over 50 years of age have on average 30% fewer spiral ganglion neurons, with losses more evident in the basal region [23, 24]. Total spiral ganglion losses increase to 50% for individuals over 80 [23]. Loss of dendritic afferents is observed in human postmortem samples, consistent with synaptic losses [14, 25]. Nonetheless, spiral ganglion neurons can still persist for decades after the onset of hearing loss, as they are frequently detected in postmortem samples from such patients [26].

Surprisingly, quantitative studies have found that human hearing metrics correlate poorly with anticipated cellular pathology. For example, while one might predict that reduced strial function should affect hearing at all frequencies, quantitative studies demonstrate that reduced strial volumes correlate strongly with high-frequency hearing loss and not with general hearing impairment [22, 27]. Similarly, a survey of older patients with high-frequency hearing loss shows that many patients retain outer hair cell activity, measured by the production of otoacoustic emissions [28]. These findings suggest that a mix of endocochlear and sensory hair cell dysfunctions may be a common mechanism in high-frequency hearing loss without specific environmental injury. Indeed, in the majority of postmortem specimens from humans with hearing loss, a combination of stria vascularis degeneration, hair cell loss, and neuronal loss is observed [22, 27, 29]. Similar findings are reported in aged non-human primates [30]. Principle component analysis of large datasets suggests that, in general, human cellular pathology in hearing loss does not naturally segregate into categories with unique cellular losses [31].

Taken together, these findings draw attention to a basic scientific question: which genes promote the survival and function of the various cells in the cochlea? Do different alleles for these genes govern a propensity to hearing loss, which varies among people? Do different genetic pathways protect different structures? If so, why are combinations of cellular damage common in older individuals with hearing loss? An interlocking network of genes regulates cellular responses to oxidative stress, metabolism, and circadian rhythm. Some genes in this network are implicated in hearing loss. However, there has not been a systematic approach to matching genetic pathways to the structures that they may protect. Once specific protective gene networks are identified, network members could become targets for therapeutics for treating hearing loss.

I propose the transcription factor Foxo3 as a candidate member for such a network that protects hearing under stress. Foxo3 is expressed in adult mouse cochlear sensory hair cells and spiral ganglion neurons [32]. In other systems, Foxo3 regulates a number of molecular pathways that promote survival and preserve function in long-lived cells. In this review, I will first provide an overview of Foxo3, its role in human longevity, and its regulation. I will describe Foxo3's role in the mouse cochlea. I will discuss recent findings that strongly implicate a role for mitochondrial function in hearing maintenance [33] in the context of Foxo3 target genes that regulate mitochondrial biogenesis and function [34]. Lastly, I will cover recent findings on how the circadian cycle affects hearing recovery after damage, in the context of Foxo3's regulation of circadian cycle [35]. Taken together, these findings indicate that cellular metabolism strongly influences the preservation of hearing, and investigations into the regulation of cochlear cellular metabolism may refine our understanding of hearing loss.

## 2. Introducing Foxo3 and Its Effects on Hearing

Foxo3 is a transcription factor of the winged helix class. It was first identified as a longevity gene in the model system* Caenorhabditis elegans *[36] and is now known as part of a metabolic network that regulates lifespan in many organisms [37]. Specific human variant alleles of Foxo3 have been linked to extreme longevity in multiple populations [38–43]. These alleles increase the amount of Foxo3 expressed in tissues [44], suggesting a protective effect.

**Figure 1:**
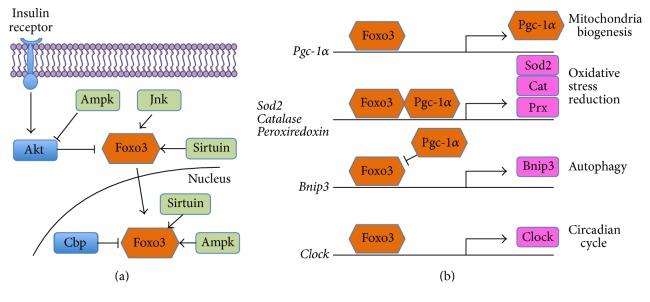
Foxo3's protein network. (a) Negative regulators (blue) of Foxo3 (orange) may act in the cytoplasm (insulin receptor, Akt) or in the nucleus (Cbp). The positive regulators (green) Ampk and sirtuins are activated by energy stress (not depicted), whereas the positive regulator (green) Jnk is activated by other stress pathways (not depicted). Sirtuins and Ampk may act in the cytoplasm or nucleus. (b) Foxo3 targets are discussed in this review. Pgc-1*α* (orange) is a transcription factor that coordinates mitochondrial biogenesis; Foxo3 and Pgc-1*α* cooperate to induce mitochondrial oxidative stress reduction genes such as Sod2, Cat, and Prx (pink). Foxo3 promotes autophagy by inducing transcription of Bnip3; here Foxo3 is antagonized by Pgc-1*α*. Lastly, Foxo3 induces Clock expression. All direct interactions shown have been demonstrated in at least one mammalian system (see text).

Foxo3 nuclear localization and transcriptional activity are regulated by a variety of extrinsic signals (Figure 1(a)). When cellular metabolic activity is low or blood sugar is high, the insulin receptor effector Akt phosphorylates Foxo3, sequestering it in the cytoplasm and blocking its activity [45–47]. When cellular metabolic activity increases, for example, during energy stress or after intense firing in neural cells, ATP levels drop and cAMP levels climb. This activates the cAMP-dependent kinase Ampk, which promotes Foxo3 transcriptional activity in two ways [48]. Transient Ampk activation inhibits Akt, allowing Foxo3 activation and nuclear localization. Under prolonged Ampk activation, Ampk localizes to the nucleus and phosphorylates Foxo3, to alter its DNA sequence binding specificity and to drive transcription of targets that promote stress resistance [49, 50]. Stress-activated Jnk, an immediate-early response kinase, can also target Foxo3 with site-specific phosphorylation that alters its binding affinity [51]. Besides phosphorylation, Foxo3 is also regulated through acetylation on lysine residues. To bind to DNA, Foxo3 requires the action of Cbp, nuclear acetylase that can open DNA for transcription through histone modification [52]. However, proximity to Cbp also enables Foxo3 acetylation, which lowers its DNA affinity. Thus, Cbp binding first initiates Foxo3-dependent transcriptional activity and then attenuates that activity. However, Foxo3 may be deacetylated by sirtuins, which, as NAD+ dependent enzymes, are activated by energy stress. In summary, Foxo3 transcriptional activity may be activated by multiple stress pathways, and each one can modulate Foxo3 DNA binding affinity to promote the expression of different targets.

Foxo3 expression in the adult mouse cochlea suggests that it acts in sensory cells. Foxo3 protein is detected in the nuclei of spiral ganglion neurons, hair cells, and pillar cells of young adult mice [32]. In contrast, Foxo3 protein was not detected in the stria vascularis [32]. Notably, Foxo3 protein becomes localized to the cytoplasm of spiral ganglion neurons as mice age. Exposure to nondamaging noise levels drives Foxo3 nuclear localization [32]. The latter observation indicates that higher cochlear activity levels promote Foxo3-dependent transcription by increasing Foxo3 concentration near DNA.

Foxo3 appears to protect hearing in mice as they age. As young adults, wild-type and *Foxo3* knockout (KO) littermates have identical hearing thresholds. However,* Foxo3*-KO mice develop high-frequency hearing loss by four months of age, whereas their wild-type littermates do not [32]. Surprisingly, inner and outer hair cells, neurons, synapses, and myelin are all present in the high-frequency turn of the* Foxo3*-KO. Outer hair cell function persists even while hearing thresholds rise, suggesting dysfunction in inner hair cells, auditory synapses, or spiral ganglion neurons. Myo7a localization in inner hair cells is affected, especially near the stereociliary bundles [32]. Synaptic size and positioning are both affected [32]. These data suggest that Foxo3 may regulate an unidentified homeostatic process required for cochlear function.

What might the data from the* Foxo3*-KO tell us about human hearing loss? First, the fact that Foxo3's location in the cell can change a few hours after nontoxic noise exposure is consistent with the idea that Foxo3 effectors maintain hearing. Second, in the older* Foxo3*-KO mice, neural components survive yet cease functioning; that is, high-frequency hearing loss develops during adulthood, but hair cells, neurons, and synapses persist. This is striking departure from central neurodegenerative diseases such as Parkinson's, where significant cellular losses precede functional losses. One might wonder if a similar defect impairs human hearing beyond the cellular pathologies discovered in human postmortem samples. Interestingly, around a quarter of postmortem samples from individuals with increased hearing thresholds display no significant cellular losses [11, 27]. Thus, there is a clinical basis for further investigation into mechanisms of cellular dysfunction in the cochlea.

## 3. Hearing Loss Can Be a Consequence of Mitochondrial Dysfunction

Mitochondria power eukaryotic life. The filamentous network of mitochondria is essential for the aerobic generation of ATP via electron transport across membranes, driven by successive redox reactions in the citric acid cycle. Incomplete reduction of the oxygen molecules contained within mitochondrial inner membrane can lead to the generation of superoxides. Cells have evolved multiple layers of enzymatic machinery to reduce superoxides and to detoxify peroxides, their derivative metabolic products. Important mechanisms include the use of glutathione as an electron donor, and mitochondrial enzymes like Sod2 and members of the peroxiredoxin family (reviewed in [53]). Under stress, these protective mechanisms can become overwhelmed, and proteins within mitochondria can become irreversibly damaged through covalent modifications by free radicals. Where this occurs, damaged parts of the mitochondrial network are no longer able to maintain the membrane voltage difference that is the hallmark of the active electron transport chain. Those parts must bud off, or fission, from the network and be targeted for destruction via autophagy [54]. The mitochondrial filamentous network grows through a process called fusion, wherein small mitochondrial fragments unite. Fusion is vital for content mixing and the maintenance of mitochondrial DNA (mtDNA) [53]. This delicate balance of destruction and renewal, of fission and fusion, has important implications for stress responses and the survival of long-lived cells.

Some of Foxo3's canonical transcriptional targets are key players in cellular processes that mitigate stress damage to mitochondria (Figure 1(b)). Foxo3 can drive transcription of the master mitochondrial regulator Pgc-1*α* [55]. Pgc-1*α* coordinates new production of mitochondrial proteins from both the nucleus and mitochondrial DNA (mtDNA) [56]. In conjunction with Pgc-1*α*, Foxo3 directly interacts with the promoters of Sod2, catalase, and peroxiredoxin to drive their expression [55, 57]. Foxo3-Pgc-1*α* interactions have been studied in the brains of Alzheimer's patients [58, 59], rodent models of Alzheimer's [60], human and rodent skeletal muscle [61, 62], human and rodent cardiac muscle [63, 64], human chondrocytes [65], rodent kidney cells [66], bovine endothelial cells [55, 67], rodent pancreas [68], and cells from human patients with mitochondrial disease [69]. Importantly, Foxo3 also promotes the expression of several autophagy proteins, which are implicated in the process that eliminates damaged organelles [70]. Foxo3 drives expression of Bnip3, which is necessary and sufficient to induce Lc3+ autophagosomes under conditions of muscle atrophy [71]. Foxo3-related autophagy studies have been performed in human and rodent skeletal muscle [71–73], human and rodent cardiomyocytes [74–77], human mesenchymal stem cells [78], human chondrocytes [75], rodent kidney cells [79], and rodent liver cells [80]. It may seem paradoxical that Foxo3 is crucial for both mitogenesis and autophagy. In studies of muscle immobilization, it appears that Pgc-1*α* and Foxo3 compete as and collaborate: overexpression of Pgc-1*α* can suppress Foxo3's ability to promote autophagy [61]. Thus, Foxo3 integrates external stress signals such as excitotoxicity and acts within a network to promote mitochondrial health. This is important because mitochondrial health is strongly implicated in the preservation of hearing.

Mutations that affect mitochondrial function adversely impact hearing as part of a spectrum of neurodegenerative disorders [54]. Mutations in six mitochondrial transfer RNA genes confer either deafness or a susceptibility to aminoglycoside-induced hearing loss [81–86]. Mitochondrial-related genes also underlie such syndromes as Kearns Sayre [87], Mohr-Tranebjaerg [88], and Wolfram [89], each of which may present with hearing loss. A number of nuclear genes that are necessary for mtDNA stability have also been identified, including SUCLA2 [90] and OPA1 [91]. Loss of function in these genes can cause early-onset human hearing loss [92, 93]. The A1555G mtDNA mutation has been shown to disrupt mitochondrial function through increased methylation of the mitochondrial 12S rRNA, a process mediated by the methyltransferase mtTFB1 [94, 95]. It has been recently shown that mice that overexpress mtTFB1 display an increase in 12S RNA methylation and subsequent activation of E2F1-dependent apoptosis in spiral ganglion cells, causing hearing loss [96].

Failure of mtDNA integrity is implicated in hearing loss during aging. Mitochondria lack the DNA repair capabilities of the nucleus. As a consequence, mitochondrial DNA accumulates somatic mutations more rapidly than nuclear DNA [97]. Notably, mutations in mitochondrial structural components encoded in mtDNA can accumulate in tissues of the elderly [98]. Such mutations can be selectively amplified from cochlear samples of presbycusis patients but not age-matched, normal hearing controls [99]. These age-related mitochondrial mutations are found in spiral ganglion neurons, organ of Corti cells, and the spiral ligament samples when isolated from sections with laser capture [100]. Thus, mitochondrial degeneration associated with aging correlates with hearing loss.

Animal models are used to determine that acute mitochondrial degradation causes hearing loss. When the cochlear mitochondrial respiratory chain is inhibited with 3-nitropropionic acid applied to the round window, permanent hearing loss ensues [101]. The predominant cellular pathology is destruction of fibrocytes in the lateral wall [102]. Mitochondrial oxidizing agents such as cisplatin and paraquat also induce cochlear degeneration [103, 104]. These data are all consistent with the idea that mitochondrial dysfunction is deleterious for hearing.

Genes that decrease mitochondrial oxidation are also implicated in preventing hearing loss from noise. Sirt3 is a mitochondrial deacetylase that regulates enzymatic activity [105–107]. Sirt3 promotes Sod2 activity [108] and regulates the mitochondrial biogenesis through deacetylation and activation of Pgc-1*α* [109]. Mice overexpressing Sirt3 have partial protection from noise damage [110]. In animal models of noise-induced hearing loss, covalent modifications of proteins due to oxidation are increased [111], and treatment with large amounts of dietary antioxidants can partly ameliorate threshold shifts from noise damage [112]. Activation of Sirt3 by administering a precursor to its activator, NAD+, also ameliorates threshold shifts from noise damage in C57BL/6 mice, which is not observed in* Sirt3*-KO mice [110]. Finally, allelic variants in the mitochondrial antioxidants paraoxonase, catalase, and SOD2 are associated with a predisposition to noise-induced hearing loss in human epidemiological studies [113–115]. All three genes decrease mitochondrial oxidative stress. Notably, Sod2 and catalase are both transcriptionally regulated by Foxo3 (Figure 1(b), [55]).

It is clear that decreasing mitochondrial oxidation is important in preventing hearing loss, as seen in genetic models, aging, acute drug studies, and noise damage. In other systems, Foxo3 governs three key elements for mitochondrial health: mitogenesis, autophagy, and the transcription of mitochondrial oxidative stress reduction proteins. Foxo3's expression in sensory cells, such as hair cells, and not in the lateral wall or stria vascularis, suggests that its protective role is restricted to mechanosensory cells rather than the cells that drive the endocochlear potential. Studies of its function in damage paradigms will be integral to understanding its role in this genetic network.

## 4. The Circadian Cycle Interacts with Hearing Recovery from Damage

The diurnal cycle governs the activity of all life. Every eukaryotic cell maintains an independent circadian cycle using two interlocking Clock mechanisms in a manner that is critical for metabolic regulation. In animals, the central nervous system synchronizes these cycles, like a pacemaker, in response to environmental cues [116].

The canonical cellular circadian cycle relies on transcriptional feedback loops to regulate protein expression. The mechanism requires positive effector proteins to drive transcription and negative effector proteins to inhibit the positive effectors. Protein levels for one positive effector, named Clock in mice, remain constant, whereas levels of another positive effector increase through the night. At the start of the day, the positive effectors together drive transcriptional expression of many genes, including the three negative regulators. It is estimated that 8–10% of transcribed genes receive regulatory input from this mechanism [117]. The negative regulators increase in quantity throughout the day. Prior to the sleep phase, the negative effectors block the positive effectors' function, which are degraded, and then the cycle begins again (reviewed in [116]). It is important to note that the loss of* Clock* function causes significant abnormalities in circadian behavior [118].

While the transcriptional mechanism of circadian cycle genes was first described decades ago in* Drosophila*, recently it was found that some metabolic functions maintain circadian cycles independently of transcription or protein synthesis [119]. NADH and NAD+ abundance rise and fall in a 24-hour cycle that governs the oxidative status of mitochondrial enzymes like peroxiredoxin [119]. In mouse liver cells, NAD+ levels increase at the start of the Clock cycle and decrease towards the end of the Clock cycle [120]. These oscillations are independent of the nutrient state of the system [120]. Analysis of the mitochondrial proteome shows that its rate-limiting metabolic proteins also vary in levels throughout the day, with their peak production occurring in the early morning [121]. When Clock protein is absent, such oscillations do not occur, significantly impairing mitochondrial function [120].

These data show that the transcriptional Clock machinery interacts with the metabolic Clock mechanism, regulating cellular metabolism, energy use, and oxidative stress response. In mouse liver cells, Foxo3 drives higher levels of* Clock* expression, promoting circadian cyclical activity [35]. Foxo3 directly binds to elements in the* Clock* promoter [35]. These findings suggest that Foxo3 regulation of the circadian cycle provides an alternative mechanism for how it maintains hearing. This is interesting because the circadian cycle has a significant impact on the extent of hearing damage from different damage paradigms.

Administration of gentamicin to humans and animals can induce hearing loss [3, 122, 123]. A recent study investigated hearing threshold shifts after daily gentamicin administration. Four groups of circadian-synchronized rats were injected with gentamicin at different times of day, spaced six hours apart from the onset of lights on. Rats receiving injections during daylight have significantly greater hearing loss [124]. It is possible that these differences could arise from circadian regulation of pharmacokinetics, diffusion of drugs to the cochlea, or liver detoxification; however, serum concentrations of gentamicin are similar among the four experimental conditions [124].

A recent report indicates that the circadian cycle also affects the cochlear response to noise, albeit in a different direction [125]. When CBA/CaJ mice are exposed to noise, they incur temporary threshold shifts [12], but only if they are exposed during the day [125]. When the same noise exposure is performed at night, the mice incur high-frequency permanent threshold shifts [125]. Given that Bdnf is sufficient to protect neurite innervation in the basal turn of the developing cochlea [126],* Bdnf* levels were investigated after noise exposure. After daytime noise exposure, cochlear* Bdnf* mRNA is upregulated over 30-fold; however, this effect is not observed after noise exposure at night [125]. In vivo treatment with an agonist to the Bdnf receptor prior to noise exposure at night prevents the permanent high-frequency threshold shifts and partially averts synaptic ribbon loss in the basal turn [125]. However, no effect of the agonist was observed when applied prior to a daytime noise exposure [125]. These data are clear evidence that the circadian cycle regulates transcriptional responses to noise exposure, which are key to hearing recovery after injury.

It is interesting to contrast noise damage to gentamicin administration with reference to the circadian cycle: for noise, exposure at night incurs greater damage, whereas, for gentamicin administration, exposure during the daytime does. These opposing papers suggest that the cochlear response to these two damage paradigms is intrinsically different and interacts with different gene pathways. It will be interesting to determine Foxo3's role in the cochlear response to hearing insults.

## 5. Concluding Remarks

The identification of genes involved in early-onset hearing loss has led to a wealth of knowledge about how the cochlea functions [127]. Identification of the genes and processes involved in the maintenance of hearing in adults will similarly lead to an even greater understanding. However, it is likely that multiple pathways interact through positive and negative feedback loops to maintain auditory function [4]. Human genetic diversity, combined with the various experiences of the human condition, may obscure the roles of individual genes and pathways in large scale human studies [4]. I speculate that different cochlear structures, such as the stria vascularis and mechanosensory cells, may rely on distinct gene pathways to promote survival and continued function. Similarly, different damage paradigms likely induce distinct responses. Strategies to improve or maintain hearing in humans thus will need to adopt a combinatorial approach, given that human pathology is prominent in multiple cochlear structures. Hence, animal genetic studies, particularly in congenic mouse strains treated with well-defined cochlear insults, may point to new mechanisms that function to maintain hearing. Identification and characterization of such mechanisms could lead to new strategies for therapies to treat hearing loss.
